# Management of suicidal cut throat injuries in a developing nation: three case reports

**DOI:** 10.1186/1757-1626-3-65

**Published:** 2010-02-22

**Authors:** Adeyi A Adoga, Nuhu D Ma'an, Henry Y Embu, Taiwo J Obindo

**Affiliations:** 1 Otorhinolaryngology Unit, Department of Surgery, Jos University Teaching Hospital, PMB 2076, Jos, Plateau State, Nigeria; 2 Department of Anesthesiology, Jos University Teaching Hospital, PMB 2076, Jos, Plateau State, Nigeria; 3 Department of Psychiatry, Jos University Teaching Hospital, PMB 2076, Jos, Plateau State, Nigeria

## Abstract

**Introduction:**

Suicidal cut throat injuries are either unreported or fortunately rare in our country. The management of these injuries requires a multi-disciplinary approach.

**Case presentations:**

This paper presents our experiences with managing three unemployed adult Nigerian males - two of Hausa ethnicity and one from the Tiv ethnic group presenting with cut throat injuries following suicidal attempts.

**Conclusion:**

The purpose of these reports is to emphasize that suicidal cut throat injuries do occur in our environment and there is a need for the collaboration of the otorhinolaryngologist, anesthesiologist and psychiatrist in the effective management of these patients. We recommend the socioeconomic improvement of individuals as a way of reducing the incidence of these injuries as unemployment was cited as a motivating factor for suicide in our patients. Ways must also be found to identify the many people in society without mental disorders who are at risk of suicidal behaviors.

## Introduction

Suicide is a known worldwide leading cause of death with psychiatric illnesses listed among the strongest predictors [1]. Other predictors listed are familial troubles and poverty [2].

There is a dearth of literature on the subject of suicidal cut throat injuries in Nigeria. It is either the cases are unreported or that they are fortunately very rare. However, literatures from other parts of the world on the prevalence of these injuries are available [2,3].

These self-inflicted injuries are obvious with transection of the hypopharynx, larynx or trachea and involvement of other parts of the body in some occasions [4]. Their initial management is straightforward and involves establishing an airway either via endotracheal intubation or tracheostomy and then surgical repair of the transected tissues [5,6], this may follow wound debridement if the wound is infected. Surgical repair is fraught with laryngo-tracheal stenosis which can be a long term morbidity suffered by patients [5].

Cut throat injuries with suicide as the motivating factor usually require rapid and interdisciplinary treatment [7]. The anesthetist and psychiatrists working in conjunction with the Otolaryngologist should manage these patients.

This paper reviews the management of three men with suicidal intent who presented to our hospital at different times within a period of 14 months with cut throat injuries.

## Case presentations

### Case 1

A 35-year-old unemployed man of the Tiv ethnic group was referred to us from the General Hospital of a neighboring state with a 12-hour history of a self-inflicted anterior neck injury. He gave inability to secure a job and fend for his family as the reason for attempting to take his life. He denied substance abuse.

On examination, we saw a conscious young man who was not in respiratory distress. He had a 14 cm anterior neck laceration involving the hypopharynx, severing the lower third of the epiglottis, exposing his laryngeal inlet with hesitant cuts on the skin of the neck. He was prepared for and had tracheostomy and primary wound closure. Tetanus prophylaxis was given to him from the referring hospital. Parenteral ceftriaxone, metronidazole and pentazocine were commenced. Nasogastric tube was passed intra-operatively following repair and removed on the 7^th ^postoperative day.

Psychiatric review revealed his act was premeditated; he woke up early hours of the morning and slit his throat in his bathroom where he was discovered by his wife at about 5 am. No prior behavioral changes were noticed by family members. There was no family history of psychiatric illness, self-injury or poisoning. His intent was to kill himself because he has been unable to provide even food for himself and family, as he had been unemployed for about 2 years. He was calm but withdrawn at review and an impression of attempted suicide by cutthroat injury in a depressed man was made. All sharps and potentially harmful objects were removed from his bedside and family members were always at his bedside to monitor him. He was commenced on setraline tablets.

Stitches were removed on the 5^th ^post-operative day. He was decannulated on the 7^th ^post-operative day with re-establishment of phonation, swallowing and breathing and discharged on the 15^th ^post-operative day.

Otolaryngologic and psychiatric follow up has been uneventful for 30 months.

### Case 2

A 27-year-old unemployed male of the Hausa ethnic group was referred from a General Hospital in a neighboring state to our Accident and Emergency unit four days following an attempted suicide with an anterior neck laceration. There was an antecedent history of depression with delusion of three years duration for which he was receiving amitriptyline tablets from the psychiatrists. No history of substance abuse.

Examination revealed a pale febrile man who was not in respiratory distress with a 12 cm transverse jagged edged anterior neck laceration exposing the hypopharynx and laryngeal inlet. The sternocleidomatoid muscles and carotid sheaths were unaffected. The wound edges were covered with slough and necrotic tissue. He was transfused two units of whole blood and had tracheostomy through which general anesthesia was administered and wound debridement and closure was done. Nasogastric tube was inserted intraoperatively following repair. Parenteral ceftriaxone and metronidazole were commenced preoperatively and continued postoperatively along with pentazocine for analgesia. He was weaned off tracheostomy with restoration of phonation and breathing on the 5^th ^postoperative day. Stitches were removed on the 5^th ^postoperative day. The nasogastric tube was removed on the 7^th ^postoperative day.

Psychiatric review revealed he had mentioned committing suicide to some family members several months before his brother discovered him with a slit throat at the back of his father's house. He had attempted poisoning himself two weeks prior to this incident stating his inability to secure a job several years after graduating from the university as the reason for the attempt. He was calm at review but withdrawn and a diagnosis of depression with cutthroat injury from attempted suicide was made and was continued on antidepressants while being monitored closely by relatives and kept from potentially harmful objects. He was discharged on the 12^th ^postoperative day.

Follow up in the clinic has been uneventful for 22 months.

### Case 3

A 55-year-old unemployed Hausa man presented to us with 24 hours history of a self-inflicted anterior neck wound from a suicidal attempt. He has 12 children from 2 wives and the unavailability of funds to cater for his family was the reason he gave for attempting to take his life. He denied substance abuse. No family history of psychiatric disorder. No history of self-injury or poisoning. He was discovered by his children in his bedroom in a pool of blood with a slit throat.

**Figure 1:**
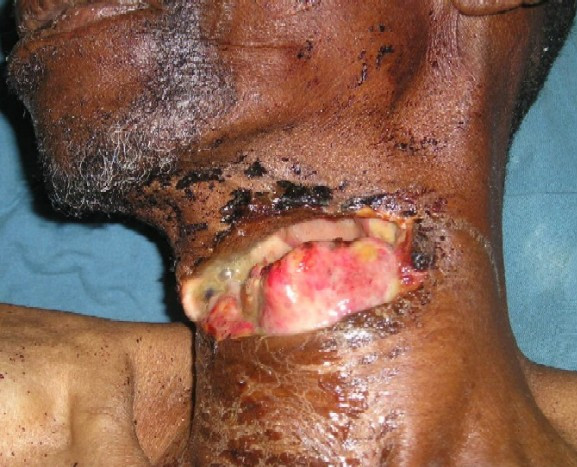
**Case 3 at presentation**.

**Figure 2:**
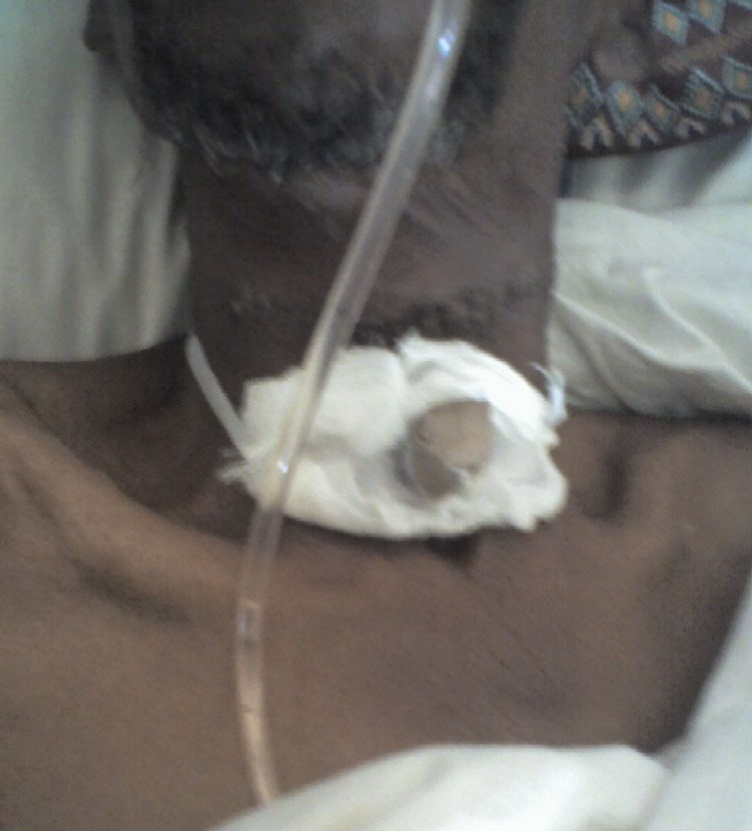
**Case 3 showing the sutured neck wound, spigotted tracheostomy tube and part of nasogastric tube in-situ**.

Examination revealed a pale middle aged man who was not dyspnoeic with a 14 cm anterior neck laceration exposing his hypopharynx and larynx (Figure 1). Tetanus prophylaxis and parenteral antibiotics were given. He was transfused 2 units of whole blood and given tracheostomy. His wound was repaired under general anesthesia administered via the tracheostomy tube. Nasogastric tube was inserted intraoperatively following repair (Figure 2). He was commenced on parenteral ciprofloxacin, metronidazole and pentazocine postoperatively. Stitches were removed and decannulation process started on the 5^th ^postoperative day and completed alongside nasogastric tube removal uneventfully on the 7^th ^postoperative day.

He started having psychiatric care and supervision immediately postoperative and was given. Follow up in the clinic has been uneventful for 6 months after discharge on the 14^th ^postoperative day.

## Discussion

The incidence of suicidal cut throat injuries in our country may be fortunately rare or the cases are unreported in literature.

When they occur, a multi disciplinary approach is required in the effective management of affected patients [7]. This requires the close collaboration of the Otolaryngologist, the anesthetist and the psychiatrist.

The anesthetist secures an uncompromised airway and makes sure the patient is breathing, the otolaryngologist assesses the injury and repairs the severed tissues with the aim of restoration of swallowing, phonation and breathing. The psychiatrist provides adequate care and supervision during and after surgical treatment.

Our patients presented without respiratory distress and the management of their airway was by executing a tracheostomy to secure a reliable airway and through which anesthetic gases were administered to effect proper surgical repair of the severed anterior neck structures under general anesthesia. However, in severe airway compromise, the airway can be maintained via endotracheal intubation [6].

Surgical repair of the severed tissues is the treatment option [8]. One patient had wound debridement with secondary suturing as a result of wound infection from late presentation, a common feature in our environment.

All our patients had injuries exposing their hypopharynx and larynx. Suturing was achieved in all of them with complete restoration of swallowing, phonation and breathing.

Suicide is one of the 10 leading causes of death in the world with more than a million deaths occurring annually [9]. It occurs 20.4 times more frequently in individuals with major depression than the general population and therefore these patients will require psychiatric intervention [10]. In a 5 year study in New Zealand of 302 individuals making medically serious suicide attempts, it was found that 6.7% died by suicide and 37% made at least one fatal suicide attempt within a 5 year period. Hence, the need for enhanced follow-up, treatment and surveillance of any individual making serious suicide attempts [11].

All our patients were considered suicidal, therefore had close psychiatric care and supervision in the immediate post-operative period and after discharge from otolaryngologic care.

Unemployment can act as a stressful life event leading to suicide [12] with studies suggesting an increase in the parasuicide and suicide rates among unemployed individuals than in the general population [13]. It is a known fact that the suicide rate among non-waged workers is significantly higher than that of waged workers [14]. The link between unemployment and mental illness is however bidirectional as individuals with mental illness are less likely to be employed than those without mental illness.

All our patients were unemployed and gave that as the motivating factor for their injuries. In our society like many others, the male is the bread winner of the family, providing for not only his immediate family but members of an extended family.

The male as the breadwinner of a family when unemployed, can be frustrated and may want to take his own life. Socioeconomic improvement of otherwise normal individuals and early detection and treatment of depression in the community is important in order to prevent serious suicide attempts. Although suicide prevention efforts should include a focus on screening and treating mental disorders, ways must also be found to identify the many people without mental disorders who are at risk of suicidal behaviors.

## Conclusion

Suicidal cut throat injuries are fortunately rare in our environment but they do occur.

A close collaboration of the Otolaryngologist, Anesthetist and Psychiatrist is required in the effective management of patients with good outcome.

Providing jobs for individuals in our country may act as confounding factors in the reduction of the prevalence of cut throat injuries of suicidal origin.

Apart from focusing on screening and the treatment of mental disorders, ways must also be found to identify the many people without mental disorders who are at risk of suicidal behaviors.

## Competing interests

The authors declare that they have no competing interests.

## Authors' contributions

AAA was the principal surgeon, performed literature search, prepared the manuscript, read and approved the final manuscript.

NDM assisted in the surgeries, postoperative management of the patients, read and approved the final manuscript.

HYE was involved in the anesthetic management of the patients, read and approved the final manuscript.

TJO was involved in the psychiatric management of the patients, read and approved the final manuscript.

## Consent

Written informed consent was obtained from the patients and their relatives for the publication of these case reports and accompanying images. Copies of the written consent are available for review by the Editor-in-Chief of this journal.
